# Socioeconomic position and subjective oral health: findings for the adult population in England, Wales and Northern Ireland

**DOI:** 10.1186/1471-2458-14-827

**Published:** 2014-08-09

**Authors:** Carol C Guarnizo-Herreño, Richard G Watt, Elizabeth Fuller, Jimmy G Steele, Jing Shen, Stephen Morris, John Wildman, Georgios Tsakos

**Affiliations:** Department of Epidemiology and Public Health, University College London, 1-19 Torrington Place, WC1E 7HB London, UK; National Centre for Social Research NatCen, 35 Northampton Square, EC1V 0AX London, UK; School of Dental Sciences, Newcastle University, Framlington Place, Tyne and Wear, NE2 4BW Newcastle Upon Tyne, UK; Institute of Health and Society, Newcastle University, Baddiley-Clark Building, Tyne and Wear, NE2 4AX Newcastle Upon Tyne, UK; Department of Applied Health Research, University College London, 1-19 Torrington Place, WC1E 7HB London, UK; Business School, Newcastle University, 5 Barrack Rd, Tyne and Wear, NE1 4SE Newcastle Upon Tyne, UK

**Keywords:** Oral health, Health inequalities, Adults, Socio-economic factors, Quality of life, Oral health-related quality of life

## Abstract

**Background:**

The objective of this study was to assess socioeconomic inequalities in subjective measures of oral health in a national sample of adults in England, Wales and Northern Ireland.

**Methods:**

We analysed data from the 2009 Adult Dental Health Survey for 8,765 adults aged 21 years and over. We examined inequalities in three oral health measures: self-rated oral health, Oral Health Impact Profile (OHIP-14), and Oral Impacts on Daily Performance (OIDP). Educational attainment, occupational social class and household income were included as socioeconomic position (SEP) indicators. Multivariable logistic regression models were fitted and from the regression coefficients, predictive margins and conditional marginal effects were estimated to compare predicted probabilities of the outcome across different SEP levels. We also assessed the effect of missing data on our results by re-estimating the regression models after imputing missing data.

**Results:**

There were significant differences in predicted probabilities of the outcomes by SEP level among dentate, but not among edentate, participants. For example, persons with no qualifications showed a higher predicted probability of reporting bad oral health (9.1 percentage points higher, 95% CI: 6.54, 11.68) compared to those with a degree or equivalent. Similarly, predicted probabilities of bad oral health and oral impacts were significantly higher for participants in lower income quintiles compared to those in the highest income level (p < 0.001). Marginal effects for all outcomes were weaker for occupational social class compared to education or income. Educational and income-related inequalities were larger among young people and non-significant among 65+ year-olds. Using imputed data confirmed the aforementioned results.

**Conclusions:**

There were clear socio-economic inequalities in subjective oral health among adults in England, Wales and Northern Ireland with stronger gradients for those at younger ages.

**Electronic supplementary material:**

The online version of this article (doi:10.1186/1471-2458-14-827) contains supplementary material, which is available to authorized users.

## Background

The association between oral health and socioeconomic position (SEP) has been well established [1–6]. Research has shown consistent inequalities with individuals in lower SEP being more likely to have poorer oral health, as measured by both clinical and subjective indicators [1–13]. Moreover, these socioeconomic inequalities frequently follow a gradient with worse oral health at successively lower socioeconomic position levels [11–17]. Similarly to inequalities in general health, the underlying causes of oral health inequalities are related to systematic social disadvantages and differential access to key resources for health [18–21]. Specifically, studies have indicated that access to material resources, knowledge-related resources and the relative position in the society play a role in the distribution of oral health [22–26].

In the UK, despite a general improvement in adult oral health during the past decades, socioeconomic inequalities in oral health persist [27, 28]. Evidence from national oral health surveys has shown consistent inequalities with social gradients in different oral health outcomes including edentulousness, decay experience, periodontal disease, and trauma [1]. Furthermore, income-related inequalities in the number of natural teeth and oral health related quality of life were found in a study using data from the 1998 UK Adult Dental Health Survey [28]. Similarly, an analysis using the English Longitudinal Survey of Aging found significant socioeconomic inequalities in edentulousness, self-perceived oral health and oral impacts on daily life for all SEP measures considered (education, income, occupational class, wealth, subjective social status, and childhood SEP) [13]. As a response to this clear and consistent evidence, tackling oral health inequalities has become a major goal of the health policy in the UK. In this context, updated information using different SEP indicators and measures of oral health is needed to support relevant public policy recommendations.

The purpose of this study was to assess socioeconomic inequalities in subjective measures of oral health and quality of life in a national sample of adults in England, Wales and Northern Ireland. The ADHS 2009 provides a unique opportunity to study these inequalities since it includes various SEP indicators and a wide range of measures of oral health and quality of life. To our knowledge, no study has performed an analysis of inequalities in subjective oral health based on the most recent data on adults’ oral health in England, Wales and Northern Ireland. Our study aimed to provide updated information using different SEP indicators and measures of oral health which is relevant to support public policy recommendations. In addition, our analyses used a comprehensive analytical approach combining regression modelling and estimation of predictive margins and conditional marginal effects. Our analysis is focused on subjective measures of oral health as we are interested in evaluating how being in different SEP level is related to 1) the general perception of oral health status and 2) the functional, social and psychological impacts of oral conditions on the quality of life of people. These subjective measures capture current rather than historic oral health and are associated with general wellbeing, unmet treatment needs and clinical outcomes. As such, they are highly relevant for planning and evaluating health services and health promotion interventions as they can influence contemporary decisions about oral health and dental care use [29–34].

## Methods

### Data source and study sample

We used data from the ADHS 2009, a survey commissioned by the NHS Information Centre and conducted by the Office for National Statistics in consortium with the National Centre for Social Research, the Northern Ireland Statistics Research Agency, and experts from UK universities. The survey had a two-stage cluster and probabilistic sample design which provides representative data at national level (England, Wales and Northern Ireland). At every eligible sampled household, all adults aged 16 years and over were invited to participate in an interview and those with at least one natural tooth were also invited to a clinical examination. The survey collected interview data from a sample of 11,380 adults, of which 6,469 completed the clinical examination. The overall household response rate was 60% and the individual response rate within households was 84%.

We limited our analyses to individuals aged 21 years and older who completed the ADHS interview. Individuals aged less than 21 years (n = 591) were not considered in our study due to the very low proportion classified in the highest educational and occupational levels compared to other participants. This presumably reflects the fact that many of the participants aged less than 21 years were still studying and as a result, comparisons based on current educational attainment and occupational social class would not be accurate.

In addition, we performed complete-case analyses, so only participants with complete information for subjective measures of oral health, SEP indicators, and all other covariates were included in the analytical sample. Therefore, from the eligible sample of 10,789 adults aged 21 years and older, we excluded 23 adults with no responses to subjective oral health outcomes, 1,999 with missing information on the SEP indicators, and 2 with missing data on any other covariate. The final sample used for our analyses was 8,765 people. Since most of the participants were excluded from the analyses because they did not have information on income (n = 1,884), we assessed the effect of these missing data on our results. For that purpose, the regression models (described below) were also estimated with data imputed using two approaches, Bayesian multiple imputation techniques, and simple regression techniques and the results were similar to those presented in this paper.

### Variables

#### Oral health outcomes

Three measures were used as outcome variables: self-rated oral health, Oral Health Impact Profile (OHIP-14), and Oral Impacts on Daily Performance (OIDP). Self-rated oral health is a summary measure that captures multiple dimensions on how people perceive their oral health status [29–32]. For this analysis, based on a 5-item scale, a binary indicator was created combining the answer options of very good/good/fair vs. bad/very bad. This categorization aimed to capture those adults with a clear negative perception about their oral health. The other two outcomes are measures of oral health-related quality of life (OHRQoL) which assess functional, social and psychological effects of oral health [35, 36]. Specifically, OHIP-14 measures the frequency of impacts of oral conditions on daily life, while OIDP also evaluates the severity of those impacts. Since in this analysis we aimed to identify cases of chronic oral impacts, we derived a binary measure for having one or more responses of ‘very often’ or ‘fairly often’ to any of the OHIP-14 items [37, 38]. For the OIDP, we were interested in assessing the presence of severe negative oral impacts and therefore, we created a binary indicator for any impact with severity rating of 3 or higher (from a scale 0 to 5).

#### Socioeconomic position measures and other covariates

We used three measures of socioeconomic position (SEP): education, occupational social class and equivalised household income. Education was measured as the highest qualification attained and was categorized into: degree or equivalent, some qualifications, or no qualifications. Occupational social class (current or most recent occupation) was assessed using the UK three-category National Statistics Socio-Economic Classification scheme (NS-SEC): managerial and professional; intermediate; and routine-manual. These three occupational categories represent broad and well differentiated employment relations and conditions of occupations in modern societies [39]. An additional category of those who never worked and long term unemployed was also considered in our analyses. When talking about social gradients, this additional category was not considered as it conceptually does not follow a hierarchical relationship with the other three categories of occupational social class. Total weekly household income was equivalised according to the McClements equivalisation scale and divided into quintiles. Age, gender, marital status, geographical location, self-rated general health and long standing illness were included as covariates given the relationship of these characteristics with oral health and SEP. In the models, age was included as a continuous variable, while the other covariates were included using the categories presented in Table 1.

**Table 1:** **Descriptive statistics for study variables, Adult Dental Health Survey 2009 (Based on a study sample of 8,765 individuals)**

Variables	n (weighted %)^a^
**Age (years)**	
21 - 34	1735 (26.65)
35 - 49	2701 (31.30)
50 - 64	2352 (23.66)
≥ 65	1977 (18.39)
**Sex**	
Male	3931 (48.44)
Female	4834 (51.56)
**Marital status**	
Single	1901 (27.05)
Married/cohabiting	5096 (54.00)
Divorced/separated	1087 (11.70)
Widowed	681 (7.26)
**Geographical location** (region)	
North England	2322 (26.17)
Midlands England	2366 (27.94)
South England (includes London)	2764 (37.46)
Wales	751 (5.29)
Northern Ireland	562 (3.14)
**Self-rated general health**	
Very good	3170 (37.00)
Good	3764 (43.50)
Fair	1363 (14.70)
Bad/very bad	468 (4.80)
**Long standing illness** (yes)	2913 (30.56)
**Educational attainment**	
Degree or equivalent	2137 (26.38)
Some educational qualifications	4909 (56.17)
No qualifications	1719 (17.44)
**Occupational social class**	
Managerial and professional	3095 (35.97)
Intermediate	1824 (19.98)
Routine and manual	3437 (39.13)
Other (never worked and long term unemployed)	409 (4.92)
**Self-rated oral health**	
Very good	2071 (23.48)
Good	4112 (46.18)
Fair	1895 (22.12)
Bad/very bad	687 (8.22)
**OHIP-14** (Fairly often or very often in at least one item)	1383 (16.04)
**OIDP** (Score of 3 or higher in any item)	1350 (15.62)

^a^Frequencies are weighted but counts are not.

### Statistical analysis

We first estimated the age-standardized prevalence of each subjective measure of oral health by the three SEP indicators. The age-standardization was performed by the direct method, using the UK population (based on the census 2011) as the standard population. Multivariable regression models were fitted to assess the relationships between subjective measures of oral health and SEP indicators. We used binary logistic regression since all oral health outcomes were dichotomous. Models were run for each combination of oral health measure (as dependent variable) and SEP indicator (as categorical explanatory variable) adjusting for age, gender, marital status, geographical location, self-rated general health, and long-standing illness. From the regression coefficients we then estimated predictive margins and conditional marginal effects to compare predicted probabilities of the outcome across different SEP levels while accounting for all other variables in the model [40, 41]. We fitted the models for the total sample and stratified by the presence of natural teeth. Among dentate participants, analyses were further stratified by age groups to evaluate if associations varied by this demographic characteristic. All models took into account the survey design and used the survey sampling probability weights to obtain population-based estimates. All analyses were conducted in Stata 12.

## Results

We analysed data for 8,765 participants of which 8,171 were dentate and 594 edentate. The sample consisted of 51.6% women, 54% married people, and 17.4% with no educational qualifications. The distributions of study variables in the analytical sample are presented in Table 1. In terms of oral health outcomes, more than two thirds of the adults rated their oral health as good or very good, and 16% reported oral impacts on daily life (Table 1). Results of age-standardized prevalence of oral health outcomes by SEP showed consistent and significant social gradients in subjective oral health by all SEP measures; namely, a higher prevalence of the outcomes at successively lower SEP levels (results not shown).

After adjusting for demographic characteristics, geographical location and general health, analyses showed significant differences in the predicted probabilities of oral health outcomes by SEP level among dentate participants. For example, the predicted probability of reporting bad oral health was 9.1 percentage points higher (95% CI: 6.54, 11.68) for participants with no qualifications compared to those with a degree or equivalent and 4.4 percentage points higher (95% CI: 3.30, 5.47) for those with some educational qualifications compared to those with a degree or equivalent (Table 2). Similarly, persons in the lower income quintiles were significantly more likely than those in the highest income level to have reported bad oral health and oral impacts after adjustment for demographic characteristics, geographical location and general health (p < 0.001). Although lower in magnitude, significant differences were also observed by occupational social class with a higher predicted probability of reporting bad oral health (5 percentage points) for subjects in manual occupations compared to those in the professional/managerial occupational level, and the same was the case for oral impacts. The predicted probabilities of oral health outcomes were significantly different from the reference category and gradually increased at lower SEP levels with only two exceptions: 1) income and self-rated oral health (not exactly graded between intermediate and second poorest quintiles of income), and 2) income and OIDP, where the predicted probabilities for the second highest and intermediate quintiles of income were not significantly different from the highest income quintile (Table 2). In addition, persons who never worked or were long term unemployed did not show significantly different probabilities of oral impacts (measured by both OHIP and OIDP) compared to those in managerial and professional occupations (Table 2). Among edentate participants, analyses indicated non-significant differences in the predicted probabilities of oral health outcomes by all SEP indicators (results not shown).

**Table 2:** **Marginal effects (differences in predicted probabilities) of subjective outcomes by SEP level, ADHS 2009, Dentate participants (n = 8,171)** ^**a**^

SEP indicator	Bad self-rated oral health	OHIP-14^b^	OIDP^c^
	Percentage points (95% CI)		
**Education**			
Degree or equivalent	Reference	Reference	Reference
Some educational qualifications	4.36 (2.95, 5.78)**	5.85 (3.82, 7.87)**	5.93 (3.88, 7.97)**
No qualifications	9.11 (6.54, 11.68)**	7.50 (4.30, 10.69)**	6.65 (3.54, 9.76)**
**Occupational social class**			
Managerial and professional	Reference	Reference	Reference
Intermediate	3.91 (1.94, 5.89)**	3.47 (0.76, 6.18)*	2.86 (0.28, 5.44)*
Routine and manual	5.06 (3.22, 6.90)**	4.64 (2.49, 6.78)**	5.14 (2.79, 7.50)**
Never worked and long term unemployed	5.94 (2.00, 9.87)**	2.21 (−3.03, 7.44)	1.38 (−2.81, 5.56)
**Equivalised Household Income**			
Wealthiest quintile	Reference	Reference	Reference
Second wealthiest quintile	2.58 (0.81, 4.35)**	3.26 (0.48, 6.04)*	2.41 (−0.27, 5.09)
Intermediate quintile	5.23 (3.32, 7.15)**	4.19 (1.38, 7.00)**	2.29 (−0.48, 5.06)
Second poorest quintile	4.81 (2.82, 6.79)**	5.15 (2.35, 7.95)**	4.58 (1.45, 7.71)**
Poorest quintile	7.43 (4.93, 9.92)**	8.43 (5.16, 11.71)**	7.07 (3.71, 10.42)**

Asterisks indicate level of significance of the marginal effects compared to the reference category (*p < 0.05, **p < 0.01).

^a^All models adjusted for demographic characteristics, geographical location and general health.

^b^Fairly often or very often in at least one item.

^c^Score of 3 or higher in any item.

Additional analyses stratified by age groups revealed that for self-rated oral health and OHIP the effect of being in the poorest income quintile or in the lowest educational category was larger among younger people (Tables 3 and 4). For the OIDP, results by age groups did not follow exactly the same pattern observed for the other two outcomes, and indicated significant and generally stronger differences by income only for participants aged 35–49 years (Table 5). For all three outcomes, the marginal effect for being in lower income levels tended to be smaller in older age groups and non-significant estimates were found among participants aged ≥ 65 years. Results by occupational social class did not show a clear pattern by age groups for any of the oral health outcomes.

**Table 3:** **Marginal effects (differences in predicted probabilities) of bad self-rated oral health by age groups and SEP level, ADHS 2009, Dentate participants (n = 8,171)** ^**a**^

SEP indicator	21 - 34 years	35 - 49 years	50 - 64 years	≥ 65 years
	Percentage points (95% CI)			
**Education**				
Degree or equivalent	Reference	Reference	Reference	Reference
Some educational qualifications	5.66 (2.77, 8.56)**	3.38 (0.75, 6.01)*	5.27 (2.36, 8.18)**	0.90 (−2.66, 4.46)
No qualifications	12.54 (5.01, 20.06)**	8.46 (3.58, 13.34)**	9.50 (5.55, 13.44)**	5.76 (1.73, 9.79)**
**Occupational social class**				
Managerial and professional	Reference	Reference	Reference	Reference
Intermediate	1.05 (−3.24, 5.33)	5.78 (2.49, 9.07)**	3.31 (−0.47, 7.09)	5.97 (2.37, 9.57)**
Routine and manual	1.78 (−1.97, 5.52)	7.25 (4.34, 10.15)**	5.14 (2.14, 8.14)**	7.80 (4.58, 11.01)**
Never worked and long term unemployed	7.67 (−0.42, 15.76)	6.00 (−0.65, 12.65)	8.44 (−1.69, 18.58)	4.46 (−0.59, 9.50)
**Equivalised Household Income**				
Wealthiest quintile	Reference	Reference	Reference	Reference
Second wealthiest quintile	4.05 (0.93, 7.16)*	3.64 (0.73, 6.55)*	−0.29 (−4.09, 3.50)	0.32 (−5.51, 6.14)
Intermediate quintile	4.92 (1.15, 8.69)*	6.99 (3.45, 10.52)**	4.52 (0.61, 8.42)*	3.06 (−2.35, 8.47)
Second poorest quintile	6.53 (1.90, 11.17)**	6.33 (2.72, 9.94)**	2.83 (−1.71, 7.36)	3.04 (−2.46, 8.54)
Poorest quintile	10.39 (5.04, 15.74)**	8.13 (4.45, 11.80)**	5.27 (0.64, 9.89)*	3.77 (−2.51, 10.05)

Asterisks indicate level of significance of the marginal effects compared to the reference category (*p < 0.05, **p < 0.01).

^a^All models adjusted for demographic characteristics, geographical location and general health.

**Table 4:** **Marginal effects (differences in predicted probabilities) of reporting “fairly often or very often” in at least one item in OHIP-14 by age groups and SEP level, ADHS 2009, Dentate participants (n = 8,171)** ^**a**^

SEP indicator	21 - 34 years	35 - 49 years	50 - 64 years	≥ 65 years
	Percentage points (95% CI)			
**Education**				
Degree or equivalent	Reference	Reference	Reference	Reference
Some educational qualifications	7.94 (4.47, 11.41)**	6.28 (3.25, 9.30)**	3.05 (−1.20, 7.31)	3.14 (−2.52, 8.80)
No qualifications	11.32 (4.38, 18.25)**	6.92 (0.63, 13.21)*	6.32 (0.76, 11.88)*	5.29 (−0.87, 11.44)
**Occupational social class**				
Managerial and professional	Reference	Reference	Reference	Reference
Intermediate	6.18 (0.61, 11.76)*	4.36 (−0.73, 9.45)	−1.74 (−5.86, 2.39)	6.87 (1.19, 12.56)*
Routine and manual	6.24 (1.80, 10.67)**	5.60 (2.01, 9.20)**	3.52 (−0.86, 7.91)	3.84 (−0.88, 8.55)
Never worked and long term unemployed	4.63 (−3.98, 13.24)	5.18 (−4.25, 14.61)	−1.74 (−10.60, 7.12)	2.35 (−5.60, 10.30)
**Equivalised Household Income**				
Wealthiest quintile	Reference	Reference	Reference	Reference
Second wealthiest quintile	5.13 (0.32, 9.94)*	2.99 (−1.29, 7.26)	1.72 (−3.78, 7.22)	2.81 (−6.41, 12.02)
Intermediate quintile	8.63 (3.60, 13.66)**	6.45 (1.08, 11.82)*	−0.29 (−5.55, 4.98)	0.81 (−6.60, 8.23)
Second poorest quintile	7.82 (1.97, 13.68)**	6.67 (1.00, 12.34)*	1.14 (−4.77, 7.05)	5.05 (−2.59, 12.69)
Poorest quintile	11.69 (5.46, 17.92)**	9.85 (4.59, 15.12)**	4.82 (−0.91, 10.55)	4.43 (−4.17, 13.03)

Asterisks indicate level of significance of the marginal effects compared to the reference category (*p < 0.05, **p < 0.01).

^a^All models adjusted for demographic characteristics, geographical location and general health.

**Table 5:** **Marginal effects (differences in predicted probabilities) of reporting a score of 3 or higher in any OIDP item by age groups and SEP level, ADHS 2009, Dentate participants (n=8,171)** ^**a**^

SEP indicator	21 - 34 years	35 - 49 years	50 - 64 years	≥ 65 years
	Percentage points (95% CI)			
**Education**				
Degree or equivalent	Reference	Reference	Reference	Reference
Some educational qualifications	5.85 (2.09, 9.61)**	6.96 (3.88, 10.14)**	5.25 (0.91, 9.57)*	3.04 (−1.83, 7.91)
No qualifications	9.32 (1.90, 16.74)*	4.42 (−1.16, 10.00)	5.19 (−0.24, 10.61)	8.17 (2.18, 14.17)**
**Occupational social class**				
Managerial and professional	Reference	Reference	Reference	Reference
Intermediate	1.40 (−3.79, 6.58)	2.30 (−1.82, 6.41)	3.47 (−1.29, 8.23)	5.85 (0.34, 11.36)*
Routine and manual	5.45 (0.64, 10.27)*	4.58 (1.26, 7.90)**	6.64 (2.12, 11.16)**	3.89 (−0.86, 8.64)
Never worked and long term unemployed	6.90 (−0.88, 14.67)	3.05 (−4.96, 11.07)	−4.42 (−11.83, 2.98)	−2.32 (−9.22, 4.59)
**Equivalised Household Income**				
Wealthiest quintile	Reference	Reference	Reference	Reference
Second wealthiest quintile	0.45 (−4.63, 5.53)	5.33 (1.49, 9.16)**	1.31 (−3.76, 6.39)	−1.89 (−10.47, 6.70)
Intermediate quintile	2.00 (−3.77, 7.76)	5.08 (0.70, 9.45)*	−0.21 (−5.04, 4.61)	−0.12 (−8.07, 7.83)
Second poorest quintile	4.24 (−1.67, 10.15)	6.53 (1.07, 11.99)*	3.82 (−2.15, 9.80)	2.49 (−5.20, 10.19)
Poorest quintile	6.19 (−0.50, 12.87)	10.58 (5.69, 15.47)**	4.65 (−1.55, 10.84)	2.34 (−6.59, 11.26)

Asterisks indicate level of significance of the marginal effects compared to the reference category (*p < 0.05, **p < 0.01).

^a^All models adjusted for demographic characteristics, geographical location and general health.

## Discussion

We examined socioeconomic inequalities in different subjective oral health measures in a representative sample of adults in England, Wales and Northern Ireland. We found inequalities in the form of social gradients among dentate, but not among edentate participants. This finding is not surprising given the previously described little variation in oral health among edentate adults and the adaptation of perceptions and expectations usually linked to becoming edentulous [13]. Further, edentate adults in our study were older, reported poorer general health and were generally more concentrated in lower SEP categories compared to the whole analytical sample (See Additional file 1: Table S1). Among dentate, important differences in the predicted probability of reporting bad oral health or oral impacts on daily life were observed by educational, occupational and income levels. Between the SEP measures used in this analysis, larger marginal effects were observed for being in lower levels of education and income compared to occupational social class. The effect of being in the poorest income quintile or in the lowest educational category was larger among young people and tended to decrease with age.

Our results suggest that subjective oral health might be related to diverse social and economic factors that influence people’s position within a society. Our measures of income, education and occupational social class represent different dimensions of SEP and therefore, the mechanisms through which they influence subjective oral health could be distinctive. First, income has been used as proxy of material-related resources and can affect oral health through access to these resources and also through its influence on self-esteem and social standing [42]. Second, education captures the knowledge-related resources of a person [43] and is a measure of life time SEP since it is generally achieved earlier in life. Education could influence subjective oral health through its effects on social and psychological resources, perceptions, and oral health related behaviours [42, 44, 45]. Finally, occupational social class refers to people’s relationship to work and to others through a society’s economic structure [46], and it may affect subjective oral health through its influence on support networks, stress in the workplace, control and autonomy. We must acknowledge, however, that as the SEP indicators used in this analysis are likely to be highly correlated with each other, these distinctive mechanisms are hypothetical and were not formally tested in this study.

Our findings on inequalities in subjective oral health agree with results of studies from different countries. In the US, analyses have shown that adults in lower income level were significantly more likely to rate their oral health as fair or poor compared to their counterparts in high income [4, 10]. Likewise, a multilevel analysis found that Australian adults with lower income were more likely to report their oral health as fair or poor even after adjusting for age, gender, education and neighbourhood socioeconomic disadvantage [6]. Another study on Australian adults revealed significant social gradients (by income, education and occupation) in social impacts from oral conditions and poor self-rated oral health [3]. Other studies also highlight the importance of relative SEP and the effects of social hierarchy on subjective oral health as the existence of social gradients has been broadly observed. In the US, based on a nationally representative sample of adults, poorer self-perceived oral health was consistently found at each lower level of education and income [9]. Even among low-income mothers in Washington State (US), self-assessed oral health was significantly worse at each lower educational and income level [47]. Poorer subjective oral health outcomes have also been found at each lower level of education, income or occupational social class in studies conducted in Sweden [14], Brazil [17] and the UK [13, 28]. In addition, some previous evidence is also in line with our finding of non-significant inequalities among edentate adults [13].

In this analysis, inequalities in subjective oral health varied according to age. We generally found larger inequalities by income and education among younger adults; for self-rated oral health and OHIP these were significant for both younger age groups while for the OIDP they were significant among 35–49 year-olds. However, for all three measures inequalities tended to fade away with age and were not significant among older adults (65+ years). Because we analysed cross-sectional data, it is difficult to disentangle if these results are explained by age effects, mortality selection, or cohort effects. If it is an age effect, our findings could indicate that material- and knowledge-related resources have a greater impact on subjective oral health in early and mid-adulthood than later in life. This stronger relationship between SEP and subjective oral health at younger ages could be explained by the “age-as-leveler” hypothesis that states that health inequalities widen in early to mid-adulthood and tend to decrease later in life [48]. One reason to explain this change is that people in higher SEP levels can only delay health decline for a specific period in their lives, and therefore, in later life they “catch up” those with low SEP. Thus, the health of persons in high SEP declines relatively slowly in early and mid-adulthood, but at older ages the rate of decline accelerates resulting in lower health inequalities [48]. Since subjective measures of oral health are significantly associated with oral health status and unmet treatment needs [29–31], the age-as-leveler hypothesis seems a sensible explanation for our results. It may also be suggested that older people in high SEP are aware of the tendency towards worse oral health with ageing and therefore may have more modest expectations about their oral health compared to earlier in life. If that is the case, lower inequalities in subjective oral health at older ages would partly be the result of adults in high SEP having 1) a faster decline in oral health status and 2) lower expectations about their oral health. Trying to explore this further, we subsequently estimated the gradients by age group for different OHIP and OIDP items. We found steeper gradients by education among the older age group in difficulty eating (measured by OIDP), potentially reflecting the worse oral health status among lower SEP groups at this age (results not shown).

Although these seem plausible interpretations of our results, we could not rule out the mortality selection and cohort effects as alternative explanations. The former refers to the fact that persons in lower SEP tend to die at younger ages, and those who survive longer have relatively better health. Then, health inequalities decrease at older ages because people with worse health at low SEP have died, leaving behind a group of relatively healthier people in low SEP levels. Mortality risk has been associated to oral health measures such as periodontal disease and number of teeth [49–52], which in turn are strongly related to subjective oral health, indicating that the mortality selection argument might also be a reasonable explanation for our results of lower inequalities at older ages.

Finally, the cohort effect refers to the fact that people in different age groups have lived under different historical contexts. These contexts could have contributed to weaker or stronger relationships between SEP and health, thereby explaining variations in health inequalities across cohorts. In this sense, people aged 65+ years represent a generation that has lived through the Second World War and the decades of economic stability and reconstruction and therefore may be a more cohesive cohort compared to younger adults that have lived primarily through a period of excessive economic growth which is linked also to wider inequalities [53]. If findings of our analyses are result of cohort effects, we could expect to see larger inequalities in the long term as the current cohort of young adults will age and the current cohort of older adults will be phased out.

This study provides comprehensive evidence on socioeconomic inequalities in subjective oral health from the most recent survey of adults’ oral health in England, Wales and Northern Ireland. It uses SEP indicators that capture different aspects of individuals’ relative position in a society. Moreover, the oral health measures considered give a comprehensive assessment of how people perceive their oral health and the effects of oral health on functional, social and psychological aspects of daily life. This study, however, has also some limitations. First, because all SEP indicators were self-reported, there could be some measurement bias. This is particularly relevant for income, as people may be reluctant to reveal accurate information [42]. The high proportion of missing income data could be the result of this unwillingness to provide such information. Second, the SEP measures used in our analyses might not be very good markers of SEP among older adults. Wealth may be a better alternative for that age group that consists primarily of pensioners, but information on wealth was not collected in this survey. Finally, the prevalence of edentulousness was very low in some SEP categories, and the lack of significant differences in predicted probabilities among edentate participants could be partially explained by these small sample sizes.

Our results have important implications for public health. The persistent social gradients in subjective oral health further highlight that health inequalities should be considered a public health priority. More importantly, the variation by age groups indicate that relevant public health interventions should primarily target younger adults as the gradients are steeper among those aged below 50 years. This is in line with adopting a life course approach and focusing earlier in life, as suggested for health inequalities in general [54]. And it may be particularly relevant for these cohorts as they are expected to keep more natural teeth and for more years than older cohorts. Therefore, achieving a good oral health status earlier in life without large differences between the different SEP strata may have a long lasting positive effect on the population’s health and well-being.

## Conclusions

In summary, our results suggest that socioeconomic inequalities in subjective oral health still exist among the UK adult population. These inequalities tend to be larger among young adults, imposing challenges for scientific knowledge and policy decision making. Further research should explore the potential contribution of specific mechanisms (material, psychosocial and behavioural) in explaining these inequalities.

## Electronic supplementary material

Additional file 1: Table S1: Descriptive statistics for study variables for edentate participants, ADHS 2009. (Based on a study sample of 594 individuals). (DOCX 39 KB)
