# Supplementary material for: Socioeconomic position and subjective oral health: findings for the adult population in England, Wales and Northern Ireland
Source: BMC Public Health. 2014 Aug 9;14:827. doi: 10.1186/1471-2458-14-827 (PMC4137102; doi:10.1186/1471-2458-14-827)
Supplement: Supplementary file 1 — Additional file 1: Table S1: Descriptive statistics for study variables for edentate participants, ADHS 2009. (Based on a study sample of 594 individuals). (DOCX 39 KB) [file 12889_2014_6946_MOESM1_ESM.docx]

Table 1a - Descriptive statistics for study variables for edentate participants, ADHS 2009

(Based on a study sample of 594 individuals)

| **Variables** | **n (weighted %)**^a^ |
| --- | --- |
| **Age (years)** |  |
| 21 - 34 | 1 (0.13) |
| 35 - 49 | 10 (2.05) |
| 50 - 64 | 109 (18.68) |
| ≥ 65 | 474 (79.14) |
| **Sex** |  |
| Male | 257 (39.02) |
| Female | 337 (60.98) |
| **Marital status** |  |
| Single | 33 (5.96) |
| Married/cohabiting | 284 (42.84) |
| Divorced/separated | 70 (11.99) |
| Widowed | 207 (39.20) |
| **Geographical location** (region) |  |
| North England | 185 (31.38) |
| Midlands England | 159 (29.07) |
| South England (includes London) | 124 (26.86) |
| Wales | 78 (8.66) |
| Northern Ireland | 48 (4.03) |
| **Self-rated general health** |  |
| Very good | 82 (13.79) |
| Good | 199 (33.38) |
| Fair | 205 (35.27) |
| Bad/very bad | 108 (17.56) |
| **Long standing illness** (yes/no) | 389 (65.06) |
| **Educational attainment** |  |
| Degree or equivalent | 11 (1.73) |
| Some educational qualifications | 215 (36.51) |
| No qualifications | 368 (61.76) |
| **Occupational social class** |  |
| Managerial and professional | 70 (12.5) |
| Intermediate | 75 (12.14) |
| Routine and manual | 384 (64.11) |
| Other (never worked and long term unemployed) | 65 (11.25) |
| **Equivalised household income**^b^ |  |
| Wealthiest quintile | 13 (2.07) |
| Second wealthiest quintile | 37 (6.42) |
| Intermediate quintile | 116 (19.13) |
| Second poorest quintile | 267 (46.51) |
| Poorest quintile | 161 (25.87) |
| **Self-rated oral health** |  |
| Very good | 2071 (25.40) |
| Good | 4112 (50.07) |
| Fair | 1895 (19.78) |
| Bad/very bad | 687 (4.75) |
| **OHIP-14** (Fairly often or very often in at least one item) | 92 (15.54) |
| **OIDP** (Score of 3 or higher in any item) | 76 (12.47) |

^a^ Frequencies are weighted but counts are not

^b^ Based on income quintiles created for the whole analytical sample
